# Evidence-based dexamethasone dosing in malignant brain tumors: what do we really know?

**DOI:** 10.1007/s11060-019-03238-4

**Published:** 2019-07-25

**Authors:** Charissa A. C. Jessurun, Alexander F. C. Hulsbergen, Logan D. Cho, Linda S. Aglio, Rishi D. S. Nandoe Tewarie, Marike L. D. Broekman

**Affiliations:** 1 Faculty of Medicine, University of Amsterdam/Amsterdam University Medical Center, Location Academic Medical Center (AMC), Meibergdreef 9, 1105 AZ Amsterdam, Noord-Holland The Netherlands; 2 Computational Neuroscience Outcomes Center (CNOC), Department of Neurosurgery, Brigham and Women’s Hospital, Harvard Medical School, 75 Francis Street, Boston, MA 02115 USA; 3 Department of Neurosurgery, Haaglanden Medical Center, Lijnbaan 32, 2512VA The Hague, Zuid-Holland The Netherlands; 4 Department of Neurosurgery, Leiden University Medical Center, Albinusdreef 2, 2333ZA Leiden, Zuid-Holland The Netherlands; 5 Brown University, 69 Brown Street, Providence, RI 02912 USA; 6 Department of Anesthesiology, Perioperative and Pain Medicine, Brigham and Women’s Hospital, Harvard Medical School, 75 Francis Street, Boston, MA 02115 USA; 7 Department of Neurology, Massachusetts General Hospital, Harvard Medical School, 55 Fruit Street, Boston, MA 02114 USA

**Keywords:** Dexamethasone, Dosing, Glioma, Brain metastases, Evidence-based medicine

## Abstract

**Purpose:**

The present study aims to conduct a systematic review of literature reporting on the dose and dosing schedule of dexamethasone (DXM) in relation to clinical outcomes in malignant brain tumor patients, with particular attention to evidence-based practice.

**Methods:**

A systematic search was performed in PubMed, Embase, Web of Science, Cochrane, Academic Search Premier, and PsycINFO to identify studies that reported edema volume reduction, symptomatic relief, adverse events and survival in relation to dexamethasone dose in glioma or brain metastasis (BM) patients.

**Results:**

After screening 1812 studies, fifteen articles were included for qualitative review. Most studies reported a dose of 16 mg, mostly in a schedule of 4 mg four times a day. Due to heterogeneity of studies, it was not possible to perform quantitative meta-analysis. For BMs, best available evidence suggests that higher doses of DXM may give more adverse events, but may not necessarily result in better clinical condition. Some studies suggest that higher DXM doses are associated with shorter survival in the palliative setting. For glioma, DXM may lead to symptomatic improvement, yet no studies directly compare different doses. Results regarding edema reduction and survival in glioma patients are conflicting.

**Conclusions:**

Evidence on the safety and efficacy of different DXM doses in malignant brain tumor patients is scarce and conflicting. Best available evidence suggests that low DXM doses may be noninferior to higher doses in certain circumstances, but more comparative research in this area is direly needed, especially in light of the increasing importance of immunotherapy for brain tumors.

**Electronic supplementary material:**

The online version of this article (10.1007/s11060-019-03238-4) contains supplementary material, which is available to authorized users.

## Background

Dexamethasone (DXM) has been a staple of neurosurgical treatment for over half a century [1]. In the context of malignant brain tumors, it is used to control peritumoral edema and alleviate symptoms due to high intracranial pressure (ICP) or focal neurologic symptoms [2]. While symptomatic improvement is usually seen within 24 to 72 h [3, 4], the use of DXM is associated with a variety of adverse events including muscular weakness, hyperglycemia, cushingoid symptoms, mental disorders, and gastrointestinal ulceration [3].

Despite the ubiquitous use of DXM for malignant brain tumors, evidence regarding the optimal dosing schedule is scarce. Doses are largely up to the discretion of treating physicians, leading to considerable practice variation [3, 5]. As a result of the ongoing advances in immunotherapy for brain tumors, the potential risks versus benefits of immunosuppressants will likely face increased scrutiny. Therefore, it is vital to address the lack of standardization of DXM dosing in the immediate future. To elucidate this question, the present study aims to conduct a systematic review of literature reporting on dose and dosing schedule of DXM in relation to clinical outcomes in glioma and brain metastasis (BM) patients and critically assess the quality of evidence in relation to this question.

## Methods

### Study design and search strategy

A systematic search was performed in PubMed, Embase, Web of Science, Cochrane, Academic Search Premier, and PsycINFO on January 18, 2019. In addition, references of included studies were checked to identify additional relevant publications. Screening and data extraction were conducted by two independent reviewers (CJ and LC). In case of disagreement over inclusion, a third reviewer (AH) was consulted. The complete search strategy can be found in Supplementary Material (S1 and S2).

### Inclusion criteria

Randomized controlled trials (RCTs), prospective or retrospective cohorts or case–control studies, and case series with > 5 patients were included. Publications reporting on the dose or dosing schedule of DXM in milligram per day (mg/day) in relation to clinical outcomes (symptomatic relief, adverse events, edema volume reduction, and survival) in patients with gliomas or BMs were included. Exclusion criteria were (1) non-human studies (2) other brain tumors including pituitary tumors and central nervous system lymphoma (3) only leptomeningeal metastases (4) combined regimens of DXM with immunotherapy or anti-emetics (5) lack of relevant outcomes (6) imprecise description of dosing schedule (e.g., doses were not standardized and only the median or range of dosing was reported) (7) no full text available, and (8) non-English publications.

### Data extraction and analysis

The following information was extracted: study characteristics including study design and sample size, patient characteristics including sex, age, baseline Karnofsky Performance Status (KPS), tumor characteristics including grade of glioma and primary tumor site for the metastases, and treatment characteristics including setting of DXM, DXM dosing and tapering schedules, and clinical outcomes. Outcomes were divided into four groups: (1) symptomatic relief (2) adverse events (3) edema volume reduction, and (4) survival. Extracted data were assessed for quantitative meta-analysis. A quality assessment of all included studies was performed based on the Cochrane Collaboration’s tool [6] for assessing risk of bias for the RCTs and the Newcastle–Ottawa Scale (NOS) [7] for the nonrandomized studies. The level of evidence was assigned using the Oxford Centre for Evidence-Based Medicine by two independent reviewers (CJ and AH) [8].

## Results

### Study selection and study characteristics

Of 1812 publications identified by systematic search, thirteen met the inclusion criteria [2, 4, 9–19]. Two additional studies were identified by reference check [20, 21] for a total of fifteen articles (see Fig. S1).

Six studies reported on > 5 glioma [2, 4, 16–19] and twelve on > 5 BM patients [4, 9–16, 19–21] (Table 2). None of the studies reported on leptomeningeal metastases. Two studies were RCTs [12, 14], one was a phase II pilot trial [21], and the rest were observational studies [2, 4, 9–11, 13, 15–20]. Four studies reported two or more DXM doses [9, 10, 12, 20], four reported dichotomized ranges [11, 14, 15, 19], and seven reported a single dose [2, 4, 13, 16–18, 21] (Table 1). Doses varied between 4 and 96 mg/day. The duration of DXM administration varied between one and 42 days. Three studies reported DXM monotherapy [9, 10, 20], two studies reported perioperative schedules [4, 18], five described peri-radiotherapy treatment [11–14, 21], and five included a combination of multiple treatment settings [2, 15–17, 19]. Ten studies, including the RCTs, were conducted in the 1970s [9, 10, 20], 1980s [15–17] and 1990s [12–14, 21] (Table 2).

**Table 1:** Study, patient and treatment characteristics

Author, year	Study type	Sample size	Critical appraisal (NOS or Cochrane risk of bias tool)	Level of evidence [8]	Percentage female patients	Mean age	KPS	Setting of treatment	Number of patients receiving DXM	DXM dose (in mg/day)	Tapering schedule (mg/day unless otherwise indicated)
Marty et al. (1973)	Observational	12	5	4	25	61	*NR*	No	10	6, 8, 12	*NR*
Fletcher et al. (1975)	Observational	8	5	4	0	58	*NR*	No	8	8, 12,16 for 27–42 days	*NR*
Graham et al. (1978)	Observational	20	5	4	35	71	*NR*	No	8	12, 16	*NR*
Pezner et al. (1982)	Observational	106	6	2b	52.8	54	*NR*	O, R	97	< 8 or > 12	*NR*
Hatam et al. (1983)	Observational	15	3	4	60	*NR*	*NR*	O, No	15	4 q.i.d. for 8–19 days	The dosage was gradually decreased from 4 q.i.d. to 1 q.i.d.. Thereafter it was gradually reduced to 1 q.d.
Muller et al. (1984)	Observational	37	4	4	*NR*	*NR*	*NR*	O, R	37	4 q.i.d. for 7 days	After 7 days the dose was reduced continuously to the maintenance dose of 4 mg/day
Weissman et al. (1991)	Observational	20	5	4	25	58	69	R	20	8 b.i.d	8 b.i.d. for 4 days, then 4 b.i.d. for 4 days, then 2 b.i.d. until final day of radiotherapy
Vecht et al. (1994)	RCT	89	Low risk of bias	1b	47.2	60	61	R	89	1, 2 or 4 q.i.d. for 28 days	16 mg: dose lowered every 4 days as follows: 3 q.i.d., 2 q.i.d., 1 q.i.d., 1 b.i.d., 1 q.d. and 0.5 q.d 8 mg: dose lowered every 4 days as follows: 1.5 q.i.d., 1 q.i.d., 1 b.i.d., 0.5 b.i.d. and 0.5 q.d 4 mg: dose lowered every 4 days as follows: 1 b.i.d., 0.5 b.i.d. and 0.5 q.d
Wolfson et al. (1994)	Phase II pilot trial	12	High risk of bias	2b	58.3	54	*NR*	R	12	All patients received 24 mg every 6 h for 48 h. After that patients were randomized between 4 q.i.d. and no DXM	*NR*
Priestman et al. (1996)	RCT; DXM dose was post-hoc analysis	533	Unclear risk of bias	2b	49.5	*NR*	*NR*	R	508	< 8 or < 8	*NR*
Tang et al. (2008)	Observational	63	7	4	55.6	62	*NR*	O, R, C, No	*NR*	> 8 or < 8	*NR*
Nguyen et al. (2013)	Observational	68	6	2b	60.3	65	70	R	68	> 16 or < 16	*NR*
Dubinski et al. (2018)	Observational	113	6	2b	48	58	80	O	113	12 mg/day until craniotomy, which was performed within 4 ± 1 day after admission. On the day of surgery all patients received a 40 mg DXM bolus, followed by 8 mg t.i.d. during the first postoperative day	*NR*
Kural et al. (2018)	Observational	28	5	4	39.3	49	*NR*	O	28	16 b.i.d. for 2 days	*NR*
Palombi et al. (2018)	Observational	459	8	2b	38.8	*NR*	*NR*	R, C	210	4 for 42 days	Alternate-day regimen of 4 mg/day (day- on/day-off) for 14 days, then alternate-day regimen of 2 mg/day for 7 days

*NOS* Newcastle–Ottawa Scale, *O* perioperative, *R* peri-radiotherapy, *C* peri-chemotherapy, *No* no other treatment, *DXM* dexamethasone, *RCT* randomized controlled trial, *KPS* Karnofsky Performance Status, *NR* not reported, *NA* not applicable, *q.i.d.* (quater in die) four times a day, *t.i.d.* (ter in die) three times a day, *b.i.d.* (bis in die) two times a day, *q.d.* (quaque die)  once a day

**Table 2:** Tumor characteristics

Author, year	Tumor location		Number of glioma patients	Grade of glioma					Number of BM patients	Primary tumor site in %					
	Infratentorial	Supratentorial		I	II	III	IV	Unknown		Breast	Lung	GI	Skin	Renal cell	Other
Marty et al. (1973)	0	9	1^d^	0	0	0	0	1	8	8.3	25	8.3	16.7	0	16.7
Fletcher et al. (1975)	0	8	1^d^	0	0	0	1	0	7	0	37.5	25	12.5	0	12.5
Graham et al. (1978)	*NR*	*NR*	8^a,d^	0	0	7	1	0	8^b^	0	62.5	12.5	0	12.5	12.5
Pezner et al. (1982)	*NR*	*NR*	0	0	0	0	0	0	106	22.6	35.8	9.4	10.4	0	32.1
Hatam et al. (1983)	*NR*	*NR*	5^c^	0	0	2	2	1	3^d^	0	33.3	0	0	33.3	33.3
Muller et al. (1984)	*NR*	*NR*	26	0	2	0	18	6	8	12.5	37.5	0	0	12.5	37.5
Weissman et al. (1991)	7	20	0	0	0	0	0	0	20	10	50	15	15	0	10
Wolfson et al. (1994)	4	12	0	0	0	0	0	0	12	25	67.7	0	0	0	8,3
Vecht et al. (1994)	*NR*	*NR*	0	0	0	0	0	0	89	24.7	48.3	9	0	9	9
Priestman et al. (1996)	*NR*	*NR*	0	0	0	0	0	0	533	18.9	58.2	0	0	0	22.9
Tang et al. (2008)	11	92	30	0	3	8	18	0	25	20	36	8	12	4	20
Nguyen et al. (2013)	*NR*	*NR*	0	0	0	0	0	0	68	23.5	48.5	7.4	1.5	7.4	11.8
Dubinski et al. (2018)	0	113	113	0	0	0	113	0	0	0	0	0	0	0	0
Kural et al. (2018)	*NR*	*NR*	21	1	9	0	11	0	7	14.3	85.7	0	0	0	0
Palombi et al. (2018)	6	453	459	0	0	0	459	0	0	0	0	0	0	0	0

*BM* brain metastasis, *GI* gastro-intestinal tract, *NR* not reported

^a^5 patients received betamethasone

^b^3 patients received betamethasone

^c^Of which one was only radiologically considered to be a glioma, no histological diagnosis was established

^d^This study was excluded for this tumor type because < 5 patients received dexamethasone

Table 1 summarizes study and patient characteristics as well as critical assessment of evidence levels. Due to the low number of included studies and the heterogeneity of doses and reported outcomes per tumor type, quantitative meta-analysis was not performed.

### Glioma

Six studies reported on the relationship between dosing of DXM and clinical outcomes in > 5 glioma patients [2, 4, 16–19]. Table 3 summarizes the results of these studies.

**Table 3:** Outcome characteristics per dose for glioma patients

Outcome category	Author	Level of evidence	Number of glioma patients/number of patients receiving DXM	Results
Symptomatic relief	Palombi et al. (2018)	2b	459/210	No DXM (n = 249) vs. DXM 4 mg/day for 42 days (n = 210), respectively: Significant difference: more symptoms in no DXM group: spatial and temporal disorientation (11.6% vs. 2.9%; p = 0.001), loss of coordination or balance (8% vs. 1.4%; p = 0.0003), altered level of consciousness (18,5% vs. 9.5%; p = 0.0013), loss of visual acuity (9.2% vs. 2.9%; p = 0.001), numbness or weakness (25.3% vs. 15.2%; p = 0.001), seizures (20.9% vs 16.2%; p = 0.001), aphasia or dysarthria (32.9% vs. 8.1%; p = 0.001) and headaches (20% vs. 13.3%; p = 0.009) No significant difference: nausea (0.8% vs. 0.5%; p = 0.5), dizziness (2% vs. 1%; p = 0.25), incontinence (0.4% vs. 0%; p = 0.31) and memory impairment (6% vs. 3.8%; p = 0.14)
Edema	Hatam et al. (1983)	4	5/5	DXM 4 mg q.i.d. for eight to nineteen days (n = 5): Edema volume pretreatment: 52 ml; edema volume posttreatment: 38.5 ml → Mean edema volume reduction of 13.5 ml (26%)
	Muller et al. (1984)	4	26/26	DXM 4 mg q.i.d. for seven days and then reduced to a maintenance dose of 4 mg/day (n = 26): Edema volume pretreatment: 20 cm^2^; edema volume posttreatment (after 20 days): 14 cm^2^ → The dimensions of edema volume did not change significantly
	Kural et al. (2018)	4	21/21	DXM 16 mg b.i.d. for two days (n = 21): Edema volume pretreatment: 3.01 ml; edema volume posttreatment: 2.96 ml → Mean edema volume reduction of 0.05 ml (1,7%) (p = 0.76)
Survival	Tang et al. (2008)	4	30/18	Low-admission DXM ( < 8 mg/day) versus high-admission DXM ( > 8 mg/day) (n = 18): DXM dose > 8 mg/day predicted shorter survival (HR 5.60, 95% CI 1.22–25.69; p = 0.027)
	Dubinski et al. (2018)	2b	113/35	12 mg DXM preoperatively (n = 35) versus no DXM preoperatively (n = 78): No significant difference in OS nor PFS was observed between the two groups (HR 1.11, 95%CI 0.74–1.66; p = 0.605 and HR 1.12, 95% CI 0.71–1.77; p = 0.605, respectively)

*DXM* dexamethasone, *OS* overall survival, *PFS* progression free survival, *q.i.d.* (quater in die) four times a day, *b.i.d.* (bis in die) two times a day

#### Symptomatic relief

One study compared glioma patients receiving chemoradiation + DXM 4 mg/day for six weeks (n = 210) to patients only receiving chemoradiation (n = 249) [2]. Patients not receiving DXM suffered significantly more neurological symptoms due to cerebral edema than patients receiving DXM, including spatiotemporal disorientation, loss of coordination or balance, altered level of consciousness, loss of visual acuity, numbness or weakness, seizures, aphasia or dysarthria and headaches. No difference between groups was observed for nausea, dizziness, incontinence or memory impairment [2].

#### Adverse events

No study reported the relationship between DXM dosing and the frequency of adverse events in glioma patients.

#### Edema

Three studies reported the effect of DXM doses on peritumoral edema [4, 16, 17]. These studies were observational studies and did not specify a cut-off point for significant volumetric edema reduction. Two studies from the 1980s [16, 17] reported on volume reduction on computed tomography (CT) imaging, while only one study reported on magnetic resonance imaging (MRI)-assessed reduction [4]. In the first CT study, glioma patients receiving DXM 4 mg four times a day (quater in die, q.i.d.) prior to surgery showed a mean edema volume reduction of 13.5 ml after eight to nineteen days of treatment (26% of the pretreatment edema volume measured on CT scan with contrast) [17]. In contrast, the other two studies in patients receiving DXM 4 mg q.i.d [16] and 16 mg two times a day (bis in die, b.i.d.) [4] showed no significant edema volume reduction after respectively 20 days and 48 h of DXM treatment.

#### Survival

Two studies reported survival in glioma patients [18, 19]. In eighteen glioblastoma patients admitted to a rehabilitation ward, a shorter survival was predicted by a high DXM dose of > 8 versus < 8 mg/day upon admission (HR 5.60, 95% CI 1.22–25.69) [19]. In the second study [18], glioblastoma patients receiving 12 mg DXM preoperatively (n = 35) and patients without preoperative DXM were compared. All patients received 40 mg perioperative DXM bolus followed by 24 mg DXM during the first postoperative day. No significant difference in overall survival or progression free survival was observed between the two groups [18].

### Brain metastases

Twelve studies [4, 9–16, 19–21] including two RCTs [12, 14] reported on the relationship between dosing of DXM and outcomes in > 5 BM patients. Results are summarized in Table 4.

**Table 4:** Outcomes per dose for brain metastasis patients

Outcome category	Author	Level of evidence	Number of BM patients/number of patients receiving DXM	Results
Symptom relief	Marty et al. (1973)	4	8/8	6 mg/day (n = 3): two patient showed neurological improvement, one showed no improvement 8 mg/day (n = 2): both patients showed neurological improvement 12 mg/day (n = 3): two patients showed neurological improvement, one showed no improvement
	Fletcher et al. (1975)	4	7/7	8 mg/day for ten days (n = 2): both patients showed neurological improvement after 10 days 12 mg/day for 2–3 weeks (n = 2): one patient showed neurological improvement after 14 days and one patient showed neurological improvement after 8 days and 17 days 16 mg/day for 7–42 days (n = 3): one patient showed neurological improvement after 6 weeks, one patient showed improvement after 12 days, and one patient showed improvement after 4 days, but then deteriorated after 9 and 14 days
	Graham et al. (1978)	4	8/5	12 mg/day (n = 1): the patient showed no neurological improvement 16 mg/day (n = 4): three patients showed neurological improvement, one patient showed no improvement
	Weissman et al. (1991)	4	20/20	16 mg/day DXM > 24 h prior to the first dose of radiation (n = 14): seven patients (50%) showed neurological improvement and seven patients (50%) had neurological stabilization. No information on neurological improvement is available for the patients receiving DXM < 24 h prior to the first dose radiation (n = 6) Fourteen patients completed the DXM treatment course as planned, three patients needed an increase in dose because of progressive neurologic symptoms, two patients showed tumor progression prompting an altered course and one patient developed hyperglycemia
	Wolfson et al. (1994)	2b	12/12	After 48 h 24 mg every 6 h i.v. (n = 12): three patients (25%) showed CR, one patient (8.3%) showed PR, 8 patients (66.7%) showed NR 4 mg every 6 h versus no DXM treatment during radiotherapy: 4 mg every 6 h (n = 7): for the post-radiotherapy change in GPS: one patient (14.3%) deteriorated, four patients (57.1%) showed no change, two patients (28.6%) showed improvement. For the post-radiotherapy change in NFC: five patients (71.4%) showed no change, one patient (14.3%) showed improvement, one patient (14.3%) deteriorated No DXM (n = 5): for the post-radiotherapy change in GPS: four patients (80%) showed no change, one patient (20%) deteriorated. The same numbers apply to the post-radiotherapy change in NFC
	Vecht et al. (1994)	1b	89/89	First series (n = 42): 8 mg/day (n = 20) versus 16 mg/day (n = 22) Day 7: 60% of the patients in the 8-mg group showed improvement in KPS compared with 54% in the 16-mg group (RR = 1.1; NS). Mean change in KPS is 8.0 (SD 10.1) versus 7.3 (SD 14.2) Day 28: 53% of the patients in the 8-mg group showed improvement in KPS compared with 81% in the 16-mg group (RR = 0.67). Mean change in KPS is 6.7 (SD 18.4) versus 13.8 (SD 14.5) Second series (n = 47): 4 mg/day (n = 24) versus 16 mg/day (n = 23): Day 7: 67% of the patients in the 4-mg group showed improvement in KPS compared with 70% in the 16-mg group (RR = 0.96; NS). Mean change in KPS is 6.7 (SD 11.3) versus 9.1 (SD 12.4) Day 28: 62% of the patients in the 4-mg group showed improvement in KPS compared with 50% in the 16-mg group (RR = 1.2; NS). Mean change in KPS is 7.1 (SD 18.2) versus 5.6 (SD 18.5)
Adverse events	Pezner et al. (1982)	2b	106/97	≥ 12 mg/day (n = 89) versus ≤ 8 mg/day (n = 8): ≥ 12 mg/day: five out of 89 patients (5.6%) developed peptic ulcer disease, six out of 89 patients (6.7%) hyperglycemia and four out of 89 patients (4.5%) steroid myopathy ≤ 8 mg/day: none of the adverse events mentioned above
	Weissman et al. (1991)	4	20/20	Five patients receiving 8 mg b.i.d. for four days developed adverse events including hyperglycemia (n = 1), candida esophagitis (n = 1), peripheral edema (n = 1), pseudo rheumatism (n = 1) and steroid withdrawal syndrome (n = 1)
	Vecht et al. (1994)	1b	89/89	4 mg/day (n = 24) versus 8 mg/day (n = 20) versus 16 mg/day (n = 45): Significant difference: the occurrence of cushingoid facies and ankle edema increased with the duration of the treatment and higher doses (p = 0.02 at day 7, and p = 0.03 at day 28) No significant difference: raised glucose, raised blood pressure, infectious disease, gastrointestinal complaints, mental changes, proximal weakness The mean KPS improvement was smaller in patients developing cushingoid facies, ankle edema or proximal weakness in comparison with patients without these symptoms. This suggests that a higher DXM dose is more effective in neurological improvement, but is associated with more adverse events which leads to a reduced net benefit on the KPS
	Nguyen et al. (2013)	2b	68/65	≥ 16 mg/day (n = 45) versus < 16 mg/day (n = 20) versus no DXM (n = 3): Patients receiving ≥ 16 mg/day DXM reported more difficulties getting to sleep (p = 0.009) and less nausea on the DSQ in comparison with patient receiving < 16 mg/day or no DXM at week 2 post-WBRT. No other items on the DSQ scale were significantly related to the DXM dose Agitation/nervousness was associated with DXM duration of ≥ 1 week (p = 0.05). No association was found between the duration ( ≤ 1 week or ≥ 1 week) of DXM treatment and other DSQ scores
Edema	Muller et al. (1984)	4	8/8	DXM 4 mg q.i.d. for seven days and then reduced to a maintenance dose of 4 mg/day (n = 8): Edema volume pretreatment: 25 cm^2^; edema volume posttreatment (after 20 days): 14 cm^2^ → mean reduction of 56% in the edema size (no p-value reported)
	Kural et al. (2018)	4	7/7	DXM 16 mg b.i.d. for two days (n = 7): Edema volume pretreatment: 1.52 ml; edema volume posttreatment: 1.57 ml → No significant reduction of the edema volume (p = 0.7)
Survival	Wolfson et al. (1994)	2b	12/12	24 mg every 6 h for 48 h and then patients were randomized to 4 mg every 6 h (n = 7) or no DXM (n = 5) during radiotherapy: Median survival of the study group of 4 months 1-year OS of the study group: 16.7%; 2-year OS of the study group: 8.3%
	Priestman et al. (1996)	2b	533/508	≤ 8 mg/day (n = 183) versus > 8 mg/day (n = 325): ≤ 8 mg/day: median survival of 96 days (95% CI 83–118); > 8 mg/day: median survival of 69 days (95% CI 61–79) (p = 0.001)
	Tang et al. (2008)	4	25/25	Low-admission DXM ( < 8 mg/day) versus high-admission DXM ( > 8 mg/day) (n = 25): DXM dose > 8 mg/day predicted poor survival (HR 4.75, 95% CI 1.41–15.98; p = 0.012)

*BM* brain metastasis, *DXM* dexamethasone, *KPS* Karnofsky performance status, *DSQ* DXM symptom questionnaire (used to assess 13 symptoms often associated with DXM toxicity), *WBRT* whole-brain radiotherapy, *NS* not significant

*q.i.d.* (quater in die) four times a day, *b.i.d.* (bis in die) two times a day

*CR* complete response, classified as the patient achieved a class 1 general performance status (*GPS*; class 1 = normal) and neurologic function class (*NFC*; able to work or to perform normal activities. Neurological findings minor or absent). *PR* partial response, classified as the patient pertained to an upgrade of the GPS and/or NFC without either worsening. *NR* nonresponse, defined as no change in both scores or a worsening of GPS and/or NFC without either improving

#### Symptomatic relief

Four small case series (5–20 patients) reported symptomatic relief, namely neurologic improvement [9, 10, 13, 20]. DXM doses ranged from 6 to 16 mg/day; the sample size of these studies limited statistical conclusions (Table 4).

One pilot prospective trial by Wolfson et al. reported twelve patients receiving 24 mg DXM intravenously every 6 h for 48 h prior to radiotherapy [21]. Three patients had complete relief of neurologic and functional symptoms, one had a partial relief, and eight experienced no relief. During radiotherapy patients were randomized in 4 mg/6 h versus no DXM. No relief of clinical or neurologic symptoms were experienced in 6/7 and 4/5 patients of the DXM and control groups, respectively.

One RCT by Vecht et al. reported the dose–effect relationship between DXM initiated seven days prior to radiotherapy and KPS in two series [12]. In the first series (n = 42), no significant difference of improvement in KPS was observed in patients receiving DXM 8 versus 16 mg/day after one week (60% vs. 54% of the patients showed improvement, respectively; non-significant (NS)). At day 28, 53% of the patients receiving 8 mg/day and 81% of the patients receiving 16 mg/day showed improvement in KPS (NS). In the second series (n = 47), no significant difference in KPS was seen in patients receiving DXM 4 mg/day in comparison with 16 mg/day at day seven and at day 28 [12].

#### Adverse events

Four studies reported adverse events in BM patients [11–13, 15]. In the RCT by Vecht et al., the incidence of cushingoid facies and ankle edema increased with the duration of treatment and with higher doses after one and four weeks (p < 0.05), while other adverse events were not significantly affected [12]. A retrospective cohort study reported on 97 patients receiving DXM ≥ 12 versus ≤ 8 mg/day started at the time of diagnosis of BMs [15]. All patients received radiation and five patients underwent surgery as initial treatment. Of the patients receiving ≥ 12 mg, 5.6% developed peptic ulcer disease, 6.7% hyperglycemia and 4.5% steroid myopathy in comparison with none of these adverse events in patients receiving ≤ 8 mg. In another series of twenty patients receiving 8 mg b.i.d. for four days, then 4 mg b.i.d. for four days and then 2 mg b.i.d. until the final day of radiotherapy, five patients developed adverse events including hyperglycemia, candida esophagitis, peripheral edema, pseudo-rheumatism, and steroid withdrawal syndrome (all n = 1) [13]. The last study prospectively reported on 68 patients undergoing palliative whole-brain radiotherapy (WBRT) [11]. Patients receiving ≥ 16 mg/day DXM reported more difficulty getting to sleep (p = 0.009) but less nausea (p-value not reported) when compared to patients receiving < 16 mg/day or no DXM two weeks after WBRT. No association was found between duration ( < 1 vs. ≥ 1 week) of DXM treatment and adverse events.

#### Edema

Two studies reported the effect of DXM doses on edema in BM patients [4, 16]. In eight patients receiving DXM 4 mg q.i.d. for seven days and then a maintenance dose of 4 mg/day prior to operation or radiotherapy, a reduction of 56% was seen in edema volume after 20 days of DXM treatment [16]. In contrast, in seven patients receiving DXM 16 mg b.i.d. for two days perioperatively, no significant reduction of the edema volume was seen (p = 0.7) [4].

#### Survival

Three studies reported survival in BM patients [14, 19]. In an RCT of 533 patients receiving two different schedules of WBRT, DXM ≤ 8 mg/day was associated with longer survival compared with > 8 mg/day (median: 96 [95% CI 83–118] vs. 69 days [95% CI 61–79] respectively; p = 0.001) [14]. Similarly, in 25 patients admitted to a rehabilitation ward after surgery, radiotherapy and/or chemotherapy, DXM dose > 8 mg/day was associated with poorer survival (HR 4.75, 95% CI 1.41–15.98; p = 0.012) [19]. Wolfson et al.’s pilot trial (n = 12) reported a median survival of 4 months.[21].

## Discussion

This systematic review aimed to assess the available evidence supporting dosing schedules of DXM for glioma and BM patients. With the exception of one RCT in BM patients [12], we found there is very little evidence to support any claim regarding the optimal dosing of DXM in malignant brain tumors. The majority of included studies, including this RCT, were conducted in the 1970s [9, 10, 20], 1980s [15–17] and 1990s [12–14, 21], predating crucial advances in diagnostic and therapeutic modalities for brain tumors.

Most studies reported a dose of 16 mg, mostly in a schedule of 4 mg q.i.d.[10, 12, 16, 17, 20]. This is congruent with a study by Sturdza et al. who reported that 45% of 34 surveyed physicians routinely prescribe DXM 4 mg q.i.d. in BM patients. The other respondents determined the dose according to the presence or absence of neurological symptoms [5].

For BMs, best available evidence suggests that higher doses of DXM may give more adverse events [11, 12, 15] but may not necessarily result in a better clinical condition (Oxford level 1b) [12]. Some studies suggest that higher doses of DXM are associated with shorter survival in a palliative setting [14, 19], but randomized studies that account for confounders, which would be necessary for causal inference, are lacking. For gliomas, less evidence is available still. While DXM may lead to symptomatic improvement [2], no studies directly compare different doses. Results regarding DXM’s effect on edema reduction [4, 16, 17] and survival [18, 19] are conflicting.

Several practice guidelines discuss dosing schedule of DXM in specific indications [22–26]; these are presented in Table 5. While these guidelines are partly based on each other, they report relatively wide ranges of starting doses (4–24 mg/day) and differ in recommendations for tapering schedules (3 days–2 weeks).

**Table 5:** Practice guidelines about dosing schedules of DXM in specific indications

Author, year	Type of tumor	Recommendations	Tapering schedule	Evidence basis	Comments
Sarin et al. (2003)	Primary tumors and metastases	Stepladder approach: the starting dose (6 mg, 12 mg, and 24 mg daily) is based on symptom severity and type of neurological symptoms and given in two divided doses: 6 mg: headache or vomiting (not severe) 12 mg: new or worsening focal deficit, with or without headache or vomiting (not severe) 24 mg + mannitol: severe headache/vomiting/altered consciousness → Dose is escalated or tapered every 48 h depending on response No DXM is recommended in the absence of symptoms of raised intracranial pressure or prophylactic use during radiotherapy	With improvement of symptoms or stabilization after every 48 h in the following steps: 24 mg, 20 mg, 16 mg, 12 mg, 8 mg, 6 mg, 4 mg, and 2 mg per day and stop	Not clearly described	
Ryken et al. (2010)	BMs	Mild symptoms: starting dose of 4–8 mg/day Moderate-severe symptoms: 16 mg/day or > No DXM is recommended in asymptomatic patients	Slow tapering over a 2 week time period, or longer in symptomatic patients depending on the individualized treatment regimen	Vecht et al. (RCT; n = 89) and Wolfson et al. (prospective cohort; n = 12) [12, 21]	This guideline was renewed in 2019 without new additions [24] These studies are also included in the present review
Kostaras et al. (2014)	High-grade glioma	Maximum of 4 mg q.i.d. postoperatively for symptomatic patients Patients who are symptomatic or have poor life expectancy can be maintained on a 0.5 - 1.0 mg/day schedule	Three schedules are proposed: Slow: 4 mg b.i.d. for 7 days, 1mg b.i.d. for 7 days, 1 mg q.d. for 7 days Fast: stop within 3 days of surgery Individualized to the patient; clinician’s discretion	Ryken’s review [23], one review [44] and four additional retrospective studies [5, 13, 45, 46]	No peer-reviewed primary studies include glioma patients Three of the original studies were not included in the present review because they did not meet inclusion criteria [5, 45, 46]
Ly et al. (2017)	Gliomas and BMs	Mild to moderate neurologic symptoms: 4 mg b.i.d. or q.d. Severe symptoms or radiographic evidence of impending herniation: an initial one-time dose of 10 mg followed by 4 mg q.i.d. Post-operatively: 16 mg/day in 2–4 doses No DXM is recommended during radiotherapy or chemotherapy unless the patient is symptomatic In patients receiving immunotherapy the administration of DXM depends on the clinical trial protocol, but it should not exceed 4 mg/day	Duration and rapidity of the tapering schedule depends on the individual patient Suggestions: Asymptomatic post-operative patients: reduce dose by 50% every 1–2 days over 5–7 days 4–8 mg/day for ≤ 2 weeks: reduce by 2 mg/day every 3 days until dose of 2 mg/day, then 1 mg/day for 3 days, then stop 8 mg/day for > 2 weeks: reduce by 2 mg/day every 5–7 days until 2 mg/day, then 1 mg/day for 5–7 days, then stop or 0.5 mg/day for several days and then stop	Kostaras’ review [25], one additional BMs review [47], and the authors’ expert opinion	No peer-reviewed primary studies include glioma patients

*DXM* dexamethasone, *BM* brain metastasis, *RCT* randomized controlled trial *q.i.d.* (quater in die) four times a day, *b.i.d.* (bis in die) two times a day, *q.d.* (quaque die) once a day

Dexamethasone dosing for other indications has been studied to varying degrees. A systematic review from 2016 [27] remarked a lack of high-quality evidence for the use of steroids in patients with metastatic spinal cord compression. The authors conclude that lower doses may be associated with similar clinical benefit and fewer adverse events when compared to higher doses. Another meta-analysis assessing impact of perioperative DXM on postoperative pain concluded that there was at best a small and clinically minimally significant dose–response relationship between DXM and pain scores [28]. A propensity score analysis of 26,634 neurosurgical patients in a national registry found preoperative steroid use to be associated with postoperative infections (odds ratio 1.38; 95% CI 1.11–1.70), even after controlling for the presence of central nervous system tumors or chemotherapy treatment [29]. In contrast, a recent Cochrane systematic review of RCTs among all surgical specialties concluded that a single dose of perioperative DXM probably does not increase the risk of surgical site infections, while there was too little evidence to draw conclusions regarding delayed wound healing [30]. Neither the national registry study nor the Cochrane review reported dose–response relations.

Strengths of this review were its strict quality assessment and evidence-based focus. Moreover, only studies providing a specific dose of DXM in correlation with the studied outcome were included. This review was extensive as it included both gliomas and BMs. The major limitation of this review lies in the heterogeneity of underlying studies. Because the included articles varied in treatment setting, outcome parameters and dose standardization, quantitative meta-analysis was not possible. Several underlying studies are relatively old or report small sample sizes. Lastly, most included studies are retrospective, and some have poor quality assessment scores. It is therefore hard to determine whether the observed outcomes in these studies were truly the consequence of different DXM doses. These limitations to the original studies support our conclusions about the lack of evidence for this widely-used treatment.

Given the widespread use of DXM in the management of malignant brain tumors, this lack of evidence regarding optimal dosing schedules is surprising. Previous practice guidelines [23, 25, 26] share the limitation that they are based on relatively few, poor-quality studies that infrequently describe dexamethasone doses in relation to outcomes that are comparable across studies. Moreover, the primary studies that they are based do not include glioma patients. Only recently have studies been published to address this question [2, 4, 18]. Level 1 evidence is not available for outcomes other than KPS improvement in patients that fit Vecht et al.’s [12] inclusion criteria. Of note, this excludes surgical patients, patients who received prior radiotherapy, patients older than 75 years, or patients with a KPS ≥ 90, among others.

The pharmacodynamics and pharmacokinetics of DXM play a relevant role in the relationship between dosing and clinical outcomes. Pitter et al. [31] demonstrated that DXM administration was an independent indicator of shorter survival in mice and humans, although doses were not specified. The authors suggest that DXM-induced antiproliferative effects may confer protection from radiotherapy and chemotherapy-induced genotoxic stress. This could explain worse survival with higher DXM doses as seen in two included studies. Individual variation in response to DXM might be explained by polymorphisms of the glucocorticoid receptor gene [32, 33]. Moreover, anticonvulsants administered to brain tumor patients (e.g. valproate, carbamazepine, phenytoin, barbiturates) could induce or inhibit cytochrome P450 liver enzymes, influencing the clearance of DXM [26]. Lastly, individual variation in plasma free fraction could cause variation in (severity of) adverse events [32].

The biologic half-life of DXM is 34–54 h, suggesting doses may not have to be dosed four times a day [34]. In one included study, a twice-daily schedule provided good clinical improvement with minimal morbidity [13]. Moreover, the cumulative dose and duration of DXM largely determines corticosteroid toxicity [32]. Given the lack of clinical evidence for q.i.d. schemes and the aforementioned biological considerations, physicians should consider a twice daily scheme as a more patient-friendly and potentially safer alternative.

The effects of steroids and immunotherapies might counteract each other [35–38]. Recent evidence has indicated that BM patients treated with immunotherapies may have diminished survival if they are concurrently receiving corticosteroids [39, 40]. Moreover, recent evidence suggests steroids may have inherent metastasis-inducing properties [41]. Therefore, it is vital to assess the role of DXM with renewed scrutiny in anticipation of this ongoing paradigm shift in cancer treatment.

While it could be argued that the benefits of DXM in brain tumor patients are so obvious that evidence-based medicine is not the most appropriate approach for this question, Sarin et al. [22] have argued that the use of steroids in traumatic brain injury had an equally formidable reputation. However, in an RCT of > 10,000 patients, steroids were found to have no benefit in this indication [42, 43].

Thus, there is a need for future investigation into dose–response relationships between DXM and several outcomes in both glioma and BM patients in different clinical settings. Studies should aim to prospectively compare different doses, dosing frequencies, and tapering schedules to determine which regimen yields the best balance between desired clinical effects and frequency of adverse events in specific patient subsets. With the current evidence in mind, the question whether lower doses are noninferior to 16 mg/day in different scenarios is particularly worthy of exploration. Moreover, the interactions between DXM and immunotherapies should be studied in neurooncological patients.

## Conclusion

Relatively little evidence is available regarding the optimal dosing scheme of DXM. In BMs, lower doses might be associated with similar efficacy and less adverse events than higher doses, but published data is heterogeneous. In gliomas, the lack of appropriate studies prevents drawing any conclusions based on more than expert opinion. The efficacy of different DXM doses is inadequately studied in the current literature; further investigation is needed to make evidence-based assessments and recommendations.

## Electronic supplementary material

Below is the link to the electronic supplementary material.

Supplementary file1 (DOCX 169 kb)
