# Supplementary material for: Evidence-based dexamethasone dosing in malignant brain tumors: what do we really know?
Source: J Neurooncol. 2019 Jul 25;144(2):249–64. doi: 10.1007/s11060-019-03238-4 (PMC6700052; doi:10.1007/s11060-019-03238-4)

**SUPPLEMENTAL MATERIAL**

**S1** | Pubmed search

(**((**("Brain Neoplasms"[Mesh] OR brain neoplasm*[tw] OR brain tumor*[tw] OR brain tumour*[tw] OR brain cancer*[tw] OR cancer of brain*[tw] OR cancer of the brain*[tw] OR intracranial neoplasm*[tw] OR cerebral neoplasm*[tw] OR cerebral tumor*[tw] OR cerebral tumour*[tw] OR cerebral cancer*[tw] OR "Glioma"[Mesh] OR glioma*[tw] OR glial cell tumor*[tw] OR glial cell tumour*[tw] OR mixed glioma*[tw] OR astrocytoma*[tw] OR glioblastoma*[tw] OR brain metasta*[tw] OR cerebral metasta*[tw]) AND ("Dexamethasone"[Mesh] OR "Dexamethasone"[tw] OR dexamethason*[tw] OR "methylfluorprednisolone"[tw] OR "hexadecadrol"[tw] OR "dexasone"[tw] OR "maxidex"[tw] OR "millicorten"[tw] OR "oradexon"[tw] OR "hexadrol"[tw] OR 9-fluoro-glucocorticoid*[tw] OR "Glucocorticoids/administration and dosage"[mesh]) AND ("administration and dosage"[Subheading] OR "Drug-Related Side Effects and Adverse Reactions"[Mesh] OR "Brain Edema"[Mesh] OR dosage*[tw] OR doze*[tw] OR dosing[tw] OR dose*[tw] OR "Drug Administration Schedule"[Mesh] OR "Drug Administration Schedule"[tw] OR "schedule"[tw] OR schedul*[tw] OR therapeutic plasma level*[tw] OR side effect*[tw] OR adverse reaction*[tw] OR adverse event*[tw] OR drug toxicit*[tw] OR brain edema*[tw] OR intracranial edema*[tw] OR cerebral edema*[tw] OR peritumor edema*[tw] OR brain oedema*[tw] OR intracranial oedema*[tw] OR cerebral oedema*[tw] OR peritumor oedema*[tw] OR edema*[tw] OR oedema*[tw] OR brain swelling*[tw] OR "Clinical Trial"[Publication Type] OR "trial"[tw] OR "RCT"[tw])) **OR** (("Brain Neoplasms"[majr] OR brain neoplasm*[ti] OR brain tumor*[ti] OR brain tumour*[ti] OR brain cancer*[ti] OR cancer of brain*[ti] OR cancer of the brain*[ti] OR intracranial neoplasm*[ti] OR cerebral neoplasm*[ti] OR cerebral tumor*[ti] OR cerebral tumour*[ti] OR cerebral cancer*[ti] OR "Glioma"[majr] OR glioma*[ti] OR glial cell tumor*[ti] OR glial cell tumour*[ti] OR mixed glioma*[ti] OR astrocytoma*[ti] OR glioblastoma*[ti] OR brain metasta*[ti] OR cerebral metasta*[ti]) AND ("Dexamethasone"[majr] OR "Dexamethasone"[ti] OR dexamethason*[ti] OR "methylfluorprednisolone"[ti] OR "hexadecadrol"[ti] OR "dexasone"[ti] OR "maxidex"[ti] OR "millicorten"[ti] OR "oradexon"[ti] OR "hexadrol"[ti] OR 9-fluoro-glucocorticoid*[ti]))**)** NOT ("Animals"[mesh] NOT "Humans"[mesh]) NOT (("Case Reports"[ptyp] OR "case report"[ti]) NOT ("case series"[ti] OR "Review"[ptyp] OR "review"[ti] OR "Clinical Study"[ptyp])))

**S2** | Embase search

(**((**(exp *"Brain Tumor"/ OR "brain neoplasm*".ti,ab OR "brain tumor*".ti,ab OR "brain tumour*".ti,ab OR "brain cancer*".ti,ab OR "cancer of brain*".ti,ab OR "cancer of the brain*".ti,ab OR "intracranial neoplasm*".ti,ab OR "cerebral neoplasm*".ti,ab OR "cerebral tumor*".ti,ab OR "cerebral tumour*".ti,ab OR "cerebral cancer*".ti,ab OR exp *"Glioma"/ OR "glioma*".ti,ab OR "glial cell tumor*".ti,ab OR "glial cell tumour*".ti,ab OR "mixed glioma*".ti,ab OR astrocytoma*.ti,ab OR glioblastoma*.ti,ab OR "brain metasta*".ti,ab OR "cerebral metasta*".ti,ab) AND (*"Dexamethasone"/ OR "Dexamethasone".ti,ab OR dexamethason*.ti,ab OR "methylfluorprednisolone".ti,ab OR "hexadecadrol".ti,ab OR "dexasone".ti,ab OR "maxidex".ti,ab OR "millicorten".ti,ab OR "oradexon".ti,ab OR "hexadrol".ti,ab OR "9-fluoro-glucocorticoid*".ti,ab OR exp *"Glucocorticoid"/do) AND (exp "drug dose"/ OR exp "Adverse Event"/ OR exp "side effect"/ OR "Brain Edema"/ OR dosage*.mp OR doze*.mp OR dosing.mp OR dose*.mp OR exp "Drug Administration"/ OR "Drug Administration Schedule".mp OR "schedule".mp OR schedul*.mp OR "therapeutic plasma level*".mp OR "side effect*".mp OR "adverse reaction*".mp OR "adverse event*".mp OR "drug toxicit*".mp OR "brain edema*".mp OR "intracranial edema*".mp OR "cerebral edema*".mp OR "peritumor edema*".mp OR "brain oedema*".mp OR "intracranial oedema*".mp OR "cerebral oedema*".mp OR "peritumor oedema*".mp OR edema*.mp OR oedema*.mp OR "brain swelling*".mp OR exp "Clinical Trial"/ OR "trial".mp OR "RCT".mp)) **OR** ((exp *"Brain Tumor"/ OR "brain neoplasm*".ti OR "brain tumor*".ti OR "brain tumour*".ti OR "brain cancer*".ti OR "cancer of brain*".ti OR "cancer of the brain*".ti OR "intracranial neoplasm*".ti OR "cerebral neoplasm*".ti OR "cerebral tumor*".ti OR "cerebral tumour*".ti OR "cerebral cancer*".ti OR exp *"Glioma"/ OR "glioma*".ti OR "glial cell tumor*".ti OR "glial cell tumour*".ti OR "mixed glioma*".ti OR "astrocytoma*".ti OR "glioblastoma*".ti OR "brain metasta*".ti OR "cerebral metasta*".ti) AND (exp *"Dexamethasone"/ OR "Dexamethasone".ti OR "dexamethason*".ti OR "methylfluorprednisolone".ti OR "hexadecadrol".ti OR "dexasone".ti OR "maxidex".ti OR "millicorten".ti OR "oradexon".ti OR "hexadrol".ti OR "9-fluoro-glucocorticoid*".ti))**)** AND exp "Humans"/ NOT (("Case Report"/ OR "case report".ti) NOT ("case series".ti OR exp "Review"/ OR "review".ti OR "Clinical Study"/ OR exp "Clinical Trial"/))) NOT (conference review or conference abstract).pt

**S3** | Flow chart of included publications


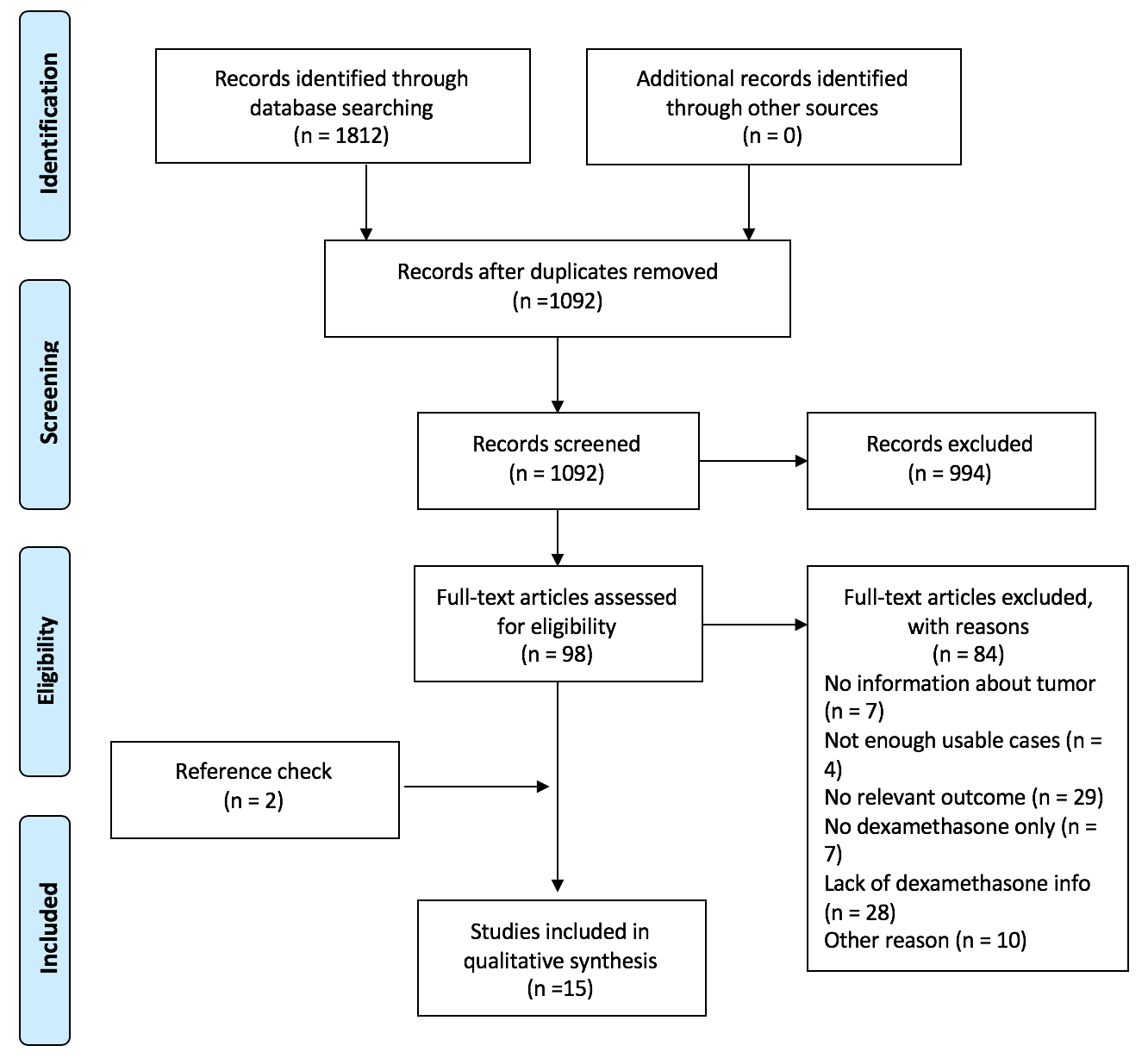

Supplement: Supplementary file 1 — Supplementary file1 (DOCX 169 kb) [file 11060_2019_3238_MOESM1_ESM.docx]
